# An automated and standardized neural index to quantify patient-ventilator interaction

**DOI:** 10.1186/cc13063

**Published:** 2013-10-16

**Authors:** Christer Sinderby, Songqiao Liu, Davide Colombo, Gianmaria Camarotta, Arthur S Slutsky, Paolo Navalesi, Jennifer Beck

**Affiliations:** 1 Keenan Research Centre in the Li Ka Shing Knowledge Institute of St-Michael’s Hospital, Toronto, Ontario, Canada; 2 Department of Critical Care, St. Michael’s Hospital, 30 Bond Street, Toronto, Ontario M5B1W8, Canada; 3 Department of Medicine and Interdepartmental Division of Critical Care Medicine, University of Toronto, Toronto, Ontario, Canada; 4 Department of Translational Medicine, Università del Piemonte Orientale “Amedeo Avogadro”, Novara, Italy; 5 Department of Pediatrics, University of Toronto, Toronto, Ontario, Canada; 6 Anesthesia and Intensive Care, Sant'Andrea Hospital, Vercelli; and CRRF Mons. L. Novarese, Moncrivello, Vercelli, Italy

## Abstract

**Introduction:**

The aim of this study was to validate an automated, objective and standardized algorithm for quantifying and displaying patient-ventilator interaction.

**Methods:**

Using a new method to detect patient-ventilator synchrony, the present study re-analyzed previously acquired and published data from 24 mechanically ventilated adult patients (Colombo et al., Crit Care Med. 2011 Nov;39(11):2452–7). Patient-ventilator interactions were evaluated by comparing ventilator pressure and diaphragm electrical activity (EAdi) waveforms, recorded during pressure support ventilation. The EAdi and ventilator pressure waveforms were analyzed for their timings (manually and automatically determined), and the error between the two waveforms was quantified. A new index of patient-ventilator interaction (NeuroSync index), which is standardized and automated, was validated and compared to manual analysis and previously published indices of asynchrony.

**Results:**

The comparison of manual and automated detection methods produced high test-retest and inter-rater reliability (Intraclass correlation coefficient = 0.95). The NeuroSync index increased the sensitivity of detecting dyssynchronies, compared to previously published indices, which were found to only detect asynchronies.

**Conclusion:**

The present study introduces an automated method and the NeuroSync index to determine patient-ventilator interaction with a more sensitive analysis method than those previously described. A dashboard-style of graphical display allows a rapid overview of patient-ventilator interaction and breathing pattern at the bedside.

## Introduction

Severe patient-ventilator asynchrony, judged by visual inspection of airway pressure and flow waveforms, can be as high as 25% in intubated and spontaneously breathing patients with acute respiratory failure, and is associated with prolonged time on mechanical ventilation and increased use of sedation [1-3]. Recently, it was shown that this pneumatic waveform analysis considerably underestimates the prevalence of asynchronies and may even fail to reveal whether or not the patient is breathing [4].

The diaphragm electrical activity (EAdi) waveform is a reliable signal to monitor the patient’s neural respiratory drive [5] as well as patient-ventilator interaction [6]. The present study aimed to introduce and to test a new objective method of automatically detecting, quantifying, and displaying patient-ventilator interaction based on the measurements of EAdi and airway pressure waveforms.

## Materials and methods

### Data

The datasets used in the present study (43 datasets in total) are from 24 adult patients with acute respiratory failure of varying etiology, intubated and on pressure support ventilation, and were obtained from previously published material from Colombo *et al*. [4]. Each patient had a nasogastric tube with a multiple array of sensors for measuring EAdi (NAVA catheter, Maquet, Solna, Sweden). The EAdi was recorded in conjunction with the ventilator pressure (P_V_) and flow waveforms during 5-minute periods. All patients were receiving mechanical ventilation with a Servoi ventilator (Maquet).

EAdi signal processing is achieved by the Edi module in the Servoi ventilator, and ensures that changes in diaphragm position along the array are accounted for [5,7] and that cardiac electrical activity is detected and replaced [5,7].

As described by Colombo *et al*. [4], waveforms of ventilator pressure and EAdi were acquired from the RS232 interface at a sampling of 100 Hz, and recorded by means of dedicated software (NAVA tracker, Maquet).

### General description of the new method to manually or automatically detect and quantify patient-ventilator interaction

Patient-ventilator interaction was evaluated by comparing the ventilator pressure and EAdi waveforms. The EAdi and ventilator pressure waveforms were analyzed with both manual and automated algorithms that detect their timings, and quantify the error between them. A new index of patient-ventilator interaction (NeuroSync index), which is standardized and automated (NeuroSync_AUTO_), was compared to manual analysis (NeuroSync_MANU_) and previously published indices of asynchrony.

### Manual analysis: detection of neural (EAdi) and ventilator time points

Two experts manually analyzed all datasets twice with a visual display of the EAdi, P_V_, and flow waveforms. Cursors were placed at the onset of EAdi (EAdi_ON_) and at about 1/3 of a decrease in EAdi from its peak (EAdi_OFF)_. This cursor placement was based on data from a separate group of patients undergoing a spontaneous breathing trial with t-piece, showing that the onset of expiratory flow coincides with a decrease in peak EAdi by about 30% (unpublished observations).

Cursors were also placed, for each breath, at the onset of P_V_ (beginning of ventilator pressurization) and end of P_V_.

### Automatic analysis

#### Detection of neural (EAdi) and ventilator time points

**Figure 1:**
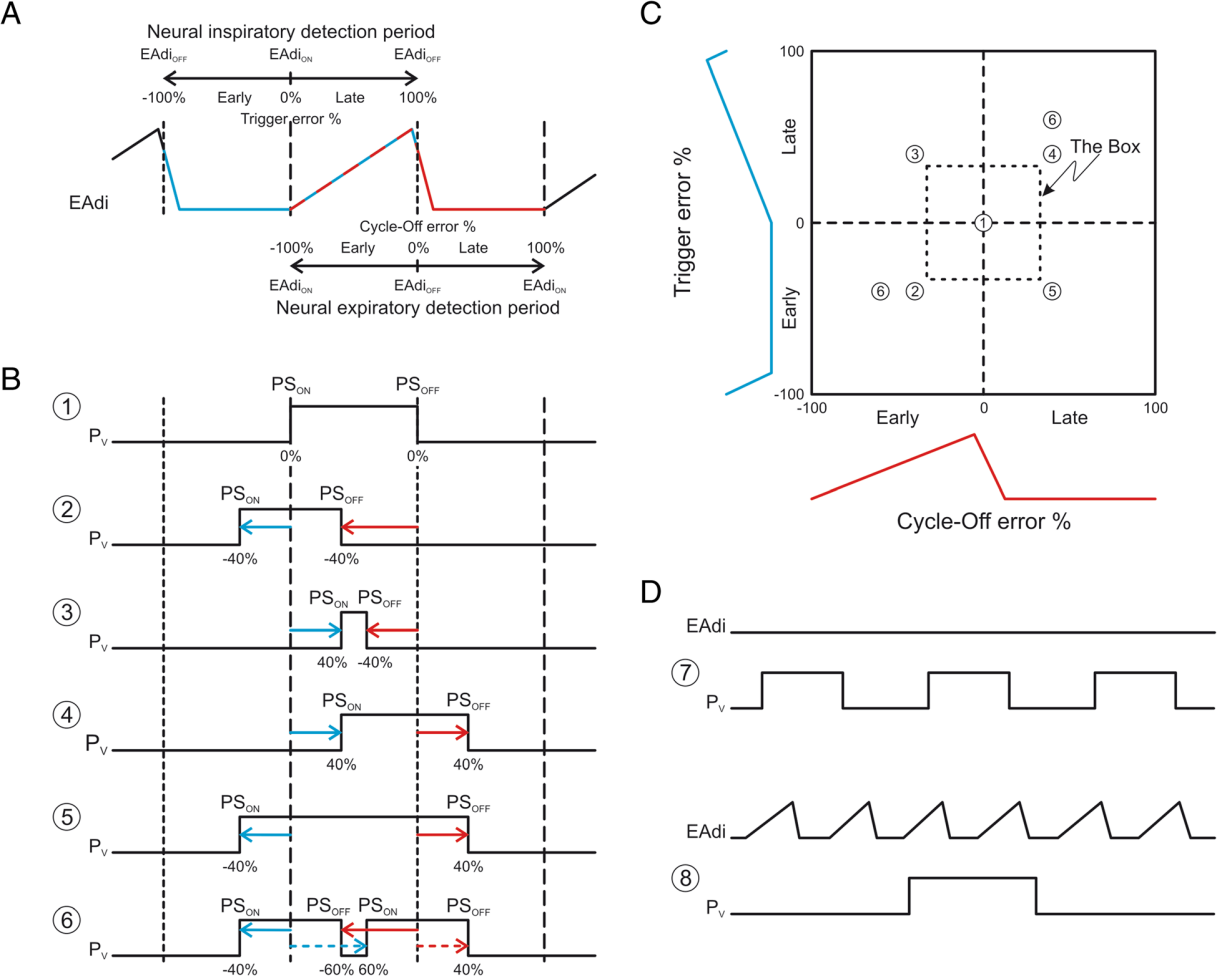
**Schematic description of the NeuroSync index and graphical display. (A)** Time tracing of a schematic electrical activity of the diaphragm (EAdi) signal. Indicators for onset (EAdi_ON_) and termination (EAdi_OFF_) are presented. Possible range of trigger error (range −100% to 100%, the neural inspiratory detection period) is indicated above the EAdi waveform. Possible range for cycle-off error (−100% to 100%, the neural expiratory detection period) is indicated below the EAdi waveform. **(B)** Time tracings of schematic ventilator pressure (P_V_) signals. Six examples of different P_V_ time tracings demonstrate different types of synchronous or dyssynchronous signal (compared to EAdi time tracing in **A)**. **(C)** Example of signals that are synchronous or dyssynchronous: graphical display. Example 1: a ventilator breath delivered in *synchrony* with EAdi: EAdi_ON_ and start of pressure delivery (PS_ON_) as well as EAdi_OFF_ and end of pressure delivery (PS_OFF_) occur simultaneously. This event has perfect synchrony (0% error) and is plotted in the center of the graphical display. Example 2: *early triggering* (PS_ON_ occurs at −40% relative to EAdi_ON_) and *early cycling-off* (PS_OFF_ at −40% relative to EAdi_OFF_). Events appear outside the box (dyssynchrony) in the lower left quadrant. Example 3: *late triggering* (PS_ON_ occurs at 40% relative to EAdi_ON_) and *early cycling-off* (PS_OFF_ at −40% relative to EAdi_OFF_). Events appear outside the box in the upper left quadrant. Example 4: *late triggering* (PS_ON_ occur at 40% relative to EAdi_ON_) and *late cycling-off* (PS_OFF_ at 40% relative to EAdi_OFF_). Events appear outside the box in the upper right quadrant. Example 5: *early triggering* (PS_ON_ occur at −40% relative to EAdi_ON_) and *late cycling-off* (PS_OFF_ at 40% relative to EAdi_OFF_). Events appear outside the box in the lower right quadrant. Example 6: multiple assist with EAdi (d*ouble triggering*). First PS assist: early triggering (PS_ON_ occurs at −40% relative to EAdi_ON_) and early cycling-off (PS_OFF_ at −40% relative to EAdi_OFF_), land outside the box in the lower left quadrant (similar to example 2). Second PS assist: late triggering (PS_ON_ occurs at 40% relative to EAdi_ON_) and late cycling-off (PS_OFF_ at 40% relative to EAdi_OFF_) land outside the box in the upper right quadrant (similar to example 4). **(D)** Example of signals that are asynchronous (that is, 100% error). Example 7 exemplifies assist without EAdi (sometimes known as auto-triggering). Example 8 illustrates EAdi without assist (also known as wasted effort) and multiple EAdi with one assist.

Automatic detection of the onset of EAdi (beginning of neural inspiration) was obtained by detecting increases in EAdi, starting from the nadir (lowest point) of the EAdi. When a preset increase in EAdi (the EAdi threshold level) was reached, the time at the nadir was stored (onset of EAdi, EAdi_ON_; Figure 1A, long-dashed vertical line). Three threshold levels were tested: 0.25, 0.5 and 1.0 μV. The amplitude at EAdi_ON_ was also stored. The EAdi_OFF_ was automatically detected by finding when the EAdi had decreased to 70% of its peak (the EAdi termination level), and this was stored as the end of EAdi (EAdi_OFF_; Figure 1A, short dashed vertical lines).

The onset of pressure support (PS_ON_) was automatically detected by searching for an increase in P_V_ >3 cm H_2_O; when reached the time value obtained at the beginning of ventilator-delivered pressurization was stored as PS_ON_ (Figure 1B, examples 1-6). The termination of pressure support (PS_OFF_; Figure 1B, examples 1-6) was automatically detected by searching for the decrease in P_V_.

#### Defragmentation

To evaluate the influence of subventilatory efforts, data were processed both with and without a defragmentation method, that is, ignoring EAdi-triggered breaths of less than 0.15 μV*s (minimal area under the EAdi curve required to be called an effort) and pressure-detected breaths of less than 1.5 cm H_2_O*s (minimal area under P_V_ curve required to be called an assist).

### Description of neural index to evaluate patient-ventilator interaction

EAdi and P_V_ timings were used to calculate an index (NeuroSync index) from both the manual (NeuroSync_MANU_) and automated (NeuroSync_AUTO_) detection methods.

Neural inspiratory detection periods were defined as segments from one detected EAdi_OFF_ to the next EAdi_OFF_. Neural expiratory detection periods were defined as segments from one EAdi_ON_ to the next EAdi_ON_ (Figure 1A).

Each neural inspiratory detection period was divided into early and late segments using the EAdi_ON_ as the divider (Figure 1A). Depending on where PS_ON_ occurred, it was expressed as a percentage of either the early or late segment of the neural inspiratory detection period (Figure 1B). Thus, an early trigger error could range between −100% and 0% (a negative trigger error) and a late trigger error could range between 0% and +100% (positive trigger error).

In the same fashion, each neural expiratory detection period was divided into early and late segments using the EAdi_OFF_ as the divider (Figure 1A). Depending on where PS_OFF_ occurred, it was expressed as a percentage of either the early or late segment of the neural expiratory detection period (Figure 1B). Thus, an early cycle-off error could range between −100% and 0% and a late cycle-off error could range between 0% and 100%.

Events where EAdi and P_V_ are completely dissociated are assigned 100% (Figure 1D).

A graphical presentation of the NeuroSync index is shown in Figure 1C. The figure depicts the neural inspiratory (y-axis) and expiratory detection (x-axis) periods and also has a box to indicate limits between synchrony (neural efforts matched to assist delivery are inside the box), dyssynchrony (neural efforts poorly related to assist delivery are outside the box), and asynchrony (neural efforts not related to assist delivery or vice versa). The limits of the box were set to ±33% difference between EAdi_ON_ and PS_ON_ as well as EAdi_OFF_ and PS_OFF_, respectively.

The NeuroSync index was calculated by averaging the absolute values of the errors for all events.

Examples of synchrony, dyssynchrony, and asynchrony are described in Figure 1.

### Comparison of indices

The NeuroSync_MANU_ and NeuroSync_AUTO_ indices were compared to the asynchrony index previously published by Colombo *et al*. (AI_Colombo_) [4]. In that paper, three examiners with specific expertise in patient-ventilator interaction used the EAdi signal to verify the accuracy of flow-pressure waveform analysis described by Thille (AI_Thille_) [1]. The asynchrony definitions used by Thille *et al*. [1] are presented in Table 1.

**Table 1:** **Asynchrony definitions for airway flow and pressure detection as described by Thille et al. **[1]**, referred to as AI**_**Thille**_

**Type of asynchrony**	**Definition**
Ineffective triggering	An abrupt airway pressure drop (≥ 0.5 cmH_2_O) simultaneous to a flow decrease (in absolute value) and not followed by an assisted cycle during the expiratory period.
Double-triggering	Two cycles separated by a very short expiratory time, defined as less than one-half of the mean inspiratory time, the first cycle being patient-triggered
Autotriggering	A cycle delivered by the ventilator without a prior airway pressure decrease, indicating that the ventilator delivered a breath that was not triggered by the patient
Short cycle	Inspiratory time less than one-half the mean inspiratory time
Prolonged cycle	Inspiratory time greater than twice the mean inspiratory time

Neural (breathing) frequency (F_N_) was calculated from the EAdi signal. Ventilator frequency (F_V_) was calculated from P_V_.

### Statistics

The intraclass correlation coefficient (ICC) was used for test-retest and inter-rater reliability (SPSS 16.0 for Windows (SPSS Inc, Chicago, IL, USA). Linear regression analysis was used to determine regression coefficients, intercepts, and determination coefficients. Unpaired comparisons were made using the Mann-Whitney rank sum test.

## Results

### Reliability of automated analysis

For the analysis of the datasets, the two expert analysts manually detected, on average, 4,562 (range 4,439 to 4,686) events (EAdi or P_V_ events). ICCs for the NeuroSync_MANU_ index obtained by the two expert analysts during two repeated analyses agreed well and are presented in Table 2.

**Table 2:** **Intraclass correlation coefficients for NeuroSync**_**MANU **_**index obtained by examiners 1 and 2 during their first and second manual analysis**

		**Examiner 2**		**Examiner 1**
		**First analysis**	**Second analysis**	**Second analysis**
**Examiner 1**	**First analysis**	0.94	0.96	0.99^a^
	**Second analysis**	0.95	0.97	
**Examiner 2**	**Second analysis**	0.97^a^		

^a^Test-retest for the same examiner.

**Figure 2:**
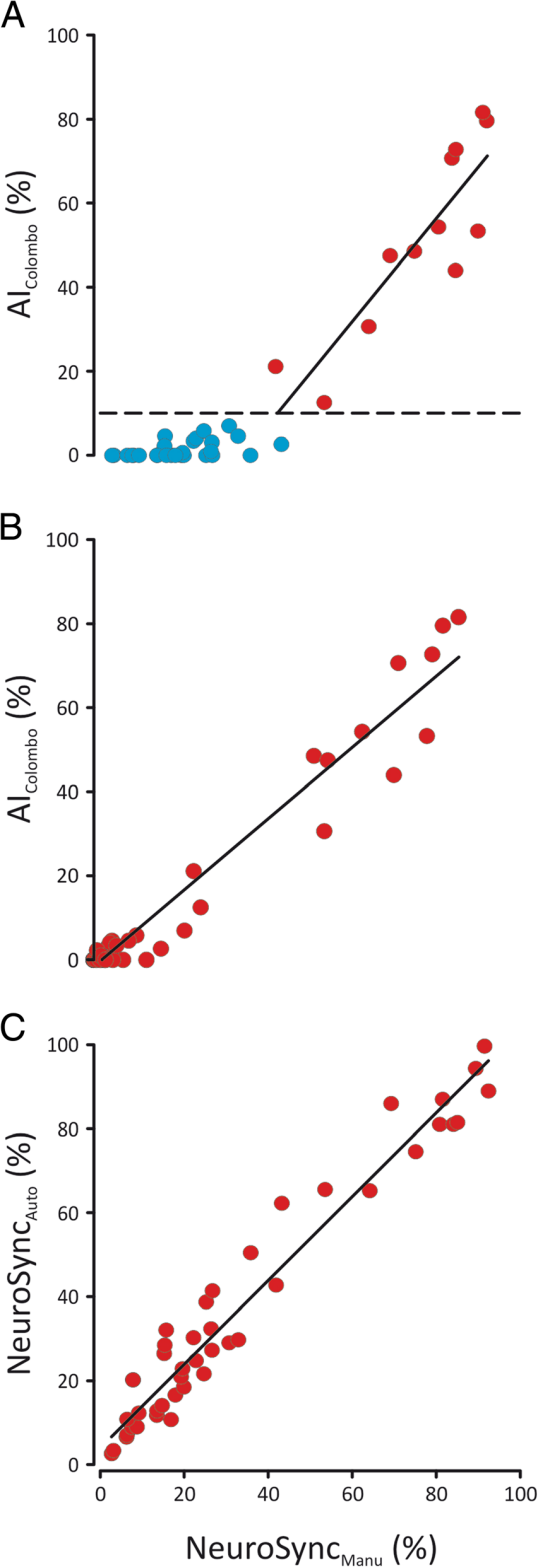
**NeuroSync**_**MANU **_**index in relation to the asynchrony index based on the definition of Colombo *et al***[4]** (AI**_**Colombo**_**) and the index for patient-ventilator interaction based on automated algorithms with automated selection of timings (NeuroSync**_**AUTO**_**)**_**. **_**(A)** Relationship between the the index for patient-ventilator interaction based on automated algorithms with manual selection of timings (NeuroSync_MANU_) (x-axis) and the EAdi-verified asynchrony index (AI_Colombo_) results published by Colombo *et al*. [4] (y-axis) for all events. Note that the first 40% increase in NeuroSync_MANU_ is not associated with any change in AI_Colombo_. After about 40% increase in NeuroSync_MANU_, the two increase in proportion. The intraclass correlation coefficient between AI_Colombo_ and NeuroSync_MANU_ for all data where the AI_Colombo_ exceeds 10% was 0.87. **(B)** Relationship between the percentage of events that were classified as asynchronous with NeuroSync_MANU_ (x-axis) and the electrical activity of the diaphragm (EAdi)-verified AI_Colombo_ (y-axis). Illustrated NeuroSync_MANU_ index and AI_Colombo_ results were obtained manually by expert analysts verifying onset and termination of inspiratory efforts by EAdi. **(C)** Relationship between NeuroSync Index calculated with either manual (x-axis) or automatic (y-axis) determination of onset and termination of EAdi.

Figure 2A demonstrates the relationship between AI_Colombo_ versus NeuroSync_MANU_ for all events and Figure 2, Panel B displays only the asynchrony events. Figure 2C shows the relationship between NeuroSync_MANU_ and NeuroSync_AUTO_ with 0.5-μV trigger threshold. Table 3 provides ICCs between the NeuroSync_MANU_ and NeuroSync_AUTO_ indices at different trigger levels, with and without defragmentation.

**Table 3:** Intraclass correlation coefficients for NeuroSync index between manual analyses (mean of four analyses by examiner 1 and 2) and automated detection

			**Manual analysis**
			**(mean of all analyses)**
Automatic analysis	Trigger level (μV)	0.25	0.91
			0.99 Defrag
		0.50	0.97
			0.95 Defrag
		1.00	0.90
			0.88 Defrag

Defrag, automated analysis excluding electrical activity of the diaphragm (EAdi)-detected breaths of less than 0.15 μV*s and pressure deflection trigger of 3.0 cm H_2_O, ignoring pressure-detected breaths of less than 1.5 cm H_2_O*s.

### Graphical display

**Figure 3:**
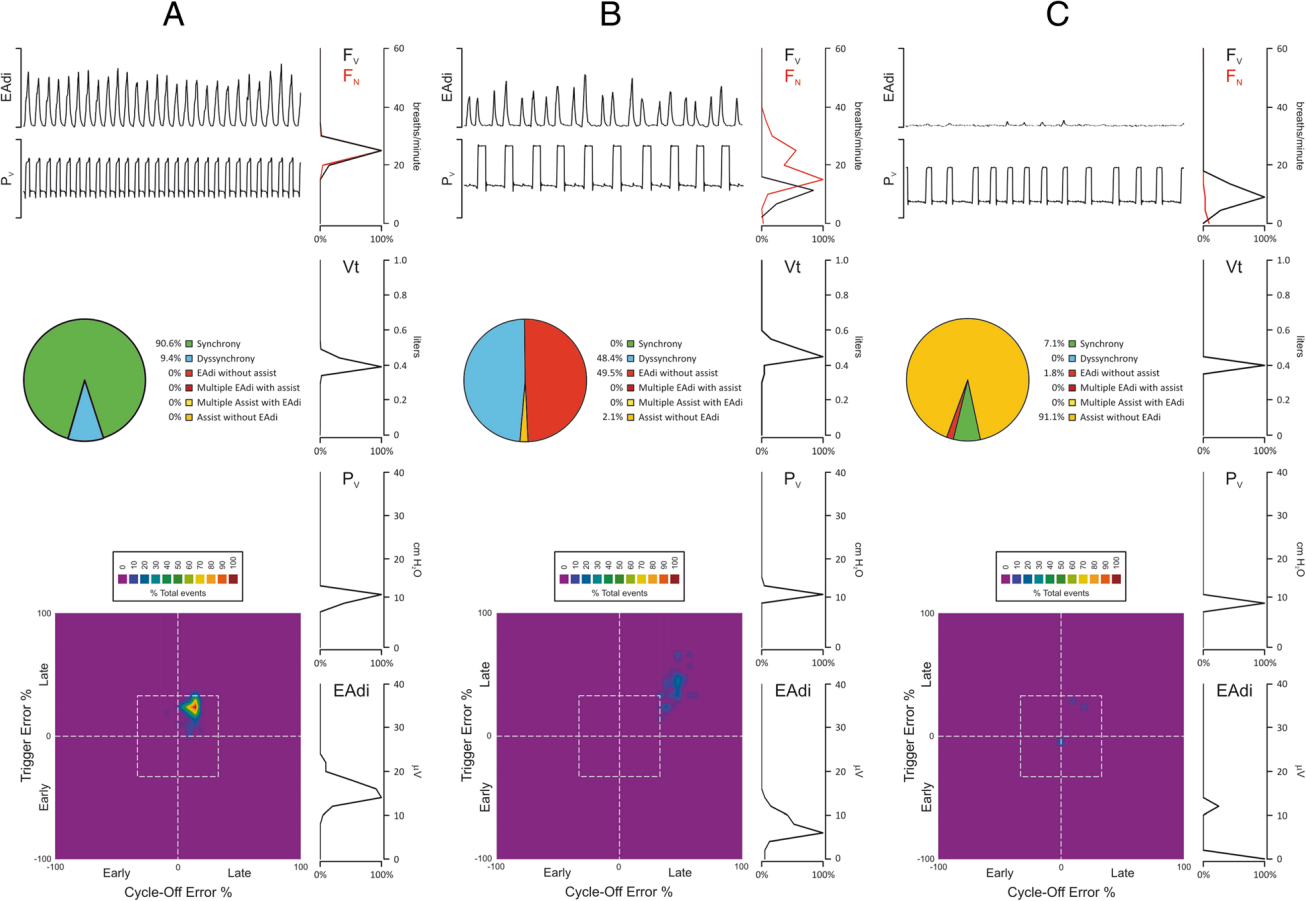
**Examples of patient-ventilator interaction and breathing pattern in three patients. (A-C)**, top (left) shows a raw tracing of electrical activity of the diaphragm (EAdi) and ventilator pressure (P_V_). Mid (left), pie-diagram shows the relative distribution of events. Bottom (left), intra-breath patient-ventilator interaction with synchrony (inside the box) and dyssynchrony (outside the box), expressed as percentage of the total number of events. Right, histograms of ventilator and neural respiratory frequency, tidal volume, P_V_ (above positive end-expiratory pressure (PEEP)), and EAdi. **(A)** Raw tracings in the top panel show clearly distinguishable EAdi and P_V_ waveforms. The pie-diagram shows that almost all breaths (close to 91%) are synchronous. It can be seen that the majority of signals appear inside the box in the upper right quadrant, indicating synchronized assist with a slightly delayed onset and termination of assist relative to the EAdi. The histograms (top to bottom on right side) show that frequency of ventilator-delivered breaths (F_V_) and of neural (patient) breaths (F_N_) are stable between 20 to 25 breaths/minute. Tidal volume was 0.4 to 0.5 l at an assist level of 12 to 14 cm H_2_O above PEEP. EAdi is concentrated in the range of 15 to 20 μV. **(B)** EAdi and P_V_ waveforms are distinguishable, but it is clear that neural efforts occur more frequently than ventilator breaths. The pie-diagram reveals 50% of the EAdi breaths were not assisted, and 2% of assist occurred without EAdi; 48% of the signals appear in the upper left quadrant outside the box, indicating substantial delays for both onset and termination of assist relative to EAdi. The histograms show that F_V_ occurs at 5 to 15 breaths/minute, whereas F_N_ demonstrates two peaks at 10 and 35 breaths/minute. Tidal volume is 0.4 to 0.6 l at an assist level of 12 to 14 cm H_2_O above PEEP. EAdi ranges from 5 to 20 μV. **(C)** The waveforms show that EAdi is infrequent and almost non-distinguishable, whereas P_V_ is clearly distinguishable and frequent. The pie-diagram indicates a 91% of assist without EAdi, 2% of EAdi without assist and only 7% of breaths land inside the box. The histograms show that F_V_ is stable at 14 to 16 breaths/minute, whereas F_N_ is either very low (few breaths) or very high (>60 breaths/minute). Tidal volume is 0.3 to 0.4 l at an assist level of 12 to 14 cm H_2_O above PEEP. EAdi shows that some breaths reach 12 to 14 μV, but the majority is lower than 2 μV.

Figure 3 provides dashboard-style graphs, displaying patient-ventilator interaction and breathing pattern in three different patients. Figure 3A demonstrates an example of good patient-ventilator interaction. Figure 3B and 3C depicts examples of poor patient-ventilator interaction.

### Subventilatory efforts

Of the analyzed datasets, 61% had ≤2 subventilatory EAdi efforts per minute and only 6% had >8 subventilatory EAdi efforts per minute. The EAdi was higher (*P* = 0.019) for datasets that had ≤2 subventilatory EAdi efforts per minute (5.7 μV, n = 26) compared to those with >2 subventilatory EAdi efforts per minute (3.0 μV, n = 17).

With respect to breathing frequency, as depicted in Table 4, the ICCs between F_N_ with automated and manual analysis were poor for breaths with EAdi amplitudes lower than 1 μV and excellent above 2 μV. Comparison of F_V_ between automated and manual analysis for defragmented breaths resulted in an ICC of 1.0.

**Table 4:** **Intraclass correlation coefficients for neural (breathing) frequency (F**_**N**_**) between manual (mean of four analyses) and automatic analysis with and without subventilatory effort defragmentation**

	**ΔEAdi**	**F**_**N**_**, automated analysis (0.5 μV + defragmentation)**
F_N_, manual analysis	All subjects	0.62
	Excluding EAdi ≤ 1 μV	0.83
	Excluding EAdi ≤ 2 μV	0.96

Determination coefficients were obtained for all subjects, and after exclusion of subjects with diaphragm electrical activity deflections (ΔEAdi) equal to or below 1 or 2 μV, respectively.

Defragmentation, automated analysis with EAdi trigger of 0.5 μV excluding EAdi-detected breaths of less than 0.15 μV*s and pressure deflection trigger of 3.0 cm H_2_O ignoring pressure-detected breaths of less than 1.5 cm H_2_O*s.

## Discussion

The present study introduces a new method for automated quantification and graphical presentation of patient-ventilator interaction and breathing pattern, using the EAdi waveform as the reference. The NeuroSync index provides better distinction for the degrees of patient-ventilator interaction by classifying events falling inside the box as synchrony, outside the box as dyssynchrony, and the remainder as asynchrony. Strong ICCs for test-retest, inter-rater, and inter-method reliability suggest that the NeuroSync index and automated detection method are both valid and reliable [9].

The NeuroSync index - in combination with the graphical illustration - allows an understanding of the *relative* differences in timing between neural efforts and assist-delivery. The index, therefore, becomes insensitive to variances in breathing pattern which can occur with age and disease. For example, a trigger delay of 100 ms in a newborn having a neural inspiratory time of 300 ms results in a 33% error, and could be considered unacceptable. However, in an adult patient with a neural inspiratory time of 800 ms, the same trigger delay represents a 12% error and could be considered acceptable. The 33% error limits may be considered big, however, delays of this magnitude are empirically common in conventional pneumatically controlled modes [8].

A neural inspiratory effort modulates motor-unit firing rate and recruitment of the diaphragm, whose temporo-spatial summation yields the EAdi [5]. Hence, the EAdi signal if acquired and processed accurately represents the neural inspiratory drive to the diaphragm. The present study uses a recommended and standardized method to process EAdi [5]. Yet, the EAdi can be disturbed by other signals such as the electrocardiograph (ECG), thus impairing accurate determination of the onset and/or end of a neural effort. For example, an ECG occurring during the onset of the neural inspiration (would be detected and replaced by its previous value) and could give a negative trigger error (airway pressure increase before EAdi increase).

The concept of a negative trigger error deserves some consideration. A negative trigger error is when the pressurization occurs prior to the onset of the EAdi signal, and can occur anywhere between the 0% and −100% error range. In our analysis, negative trigger errors that were less than −33% error (that is, 0% to −33%) were classified as synchrony. When could this possibly be observed? As mentioned above, the ECG replacement algorithm could interfere with detection of the onset of the EAdi. In the present EAdi analysis, we estimated the maximum error to determine the onset and end of a single EAdi effort to be equivalent to the duration of P- or QRS-waveforms. When averaged over hundreds of breaths, this error would become minute.

Negative trigger errors falling between the −33% and −100% error were classified as dyssynchrony. Although one could consider a significant negative trigger error as an asynchronous event, we quantified them and classified them as dyssynchrony in order to provide symmetry of our numerical and graphical representation. In the present study, these events were rare: only 0.9% of the total number of events fell into the category of negative trigger error and were classified as dyssynchrony (data not shown). Recent work by Akoumianaki *et al*. [9] in sedated and mechanically ventilated patients demonstrated respiratory entrainment, where patients’ neural efforts shortly follow ventilator pressurization. In theory, if this time delay would fall between −33 and −100% negative trigger error, it would be classified as dyssynchrony.

Naturally, extreme negative trigger errors are actually assist-without-Eadi and were classified as asynchrony, and given an error of 100%, as there is no patient interaction (no effort) associated with the ventilator.

Our analysis showed that subventilatory efforts are rare and typically related to very low EAdi amplitudes (<2 to 3 μV) and that their elimination has its greatest value at sensitive trigger/threshold levels (0.25 μV). A problem of subventilatory EAdi efforts is that if they fail to initiate assist, the event is classified as EAdi-without-assist (ineffective effort, also known as wasted effort), whereas if assist is initiated it is classified as assist-without-EAdi (auto-triggering). Subventilatory EAdi efforts introduced uncertainties in determining neural breathing pattern and with regards to the agreement between manual and automated analysis to determine F_N_, it was clear that low EAdi amplitude worsened the reliability. This underlines the importance of a good signal to noise ratio for automated analysis. It must be noted that very low or no EAdi signal that does not reach the EAdi threshold does not preclude that patients may be activating inspiratory muscles other than the diaphragm. The nature of these subventilatory efforts is unknown, but is most likely related to spontaneous firing or recruitment of diaphragm motor units. This is to be distinguished from signal disturbances, which are typically much higher in amplitude, and managed by other software algorithms.

### Comparison of NeuroSync definitions to previous asynchrony definitions

The group of Thille *et al*. [1] was the first to describe and quantify major *asynchronies*, such as wasted efforts and auto-triggering, using only airway pressure and flow waveforms, albeit without the EAdi as a reference.

The NeuroSync event defined as EAdi-without-assist corresponds to ineffective triggering with the AI_Thille_. An inspiratory effort not rewarded by a ventilator breath is a failure for a triggered mode and is the asynchrony predominantly associated with adverse patient outcomes [1,2]. As ineffective triggering typically relates to a failure of the conventional ventilator flow and pressure sensors to detect an inspiratory effort, it is not surprising that the prevalence of ineffective triggering is greatly underestimated by airway flow and pressure detection [4].

The NeuroSync event defined as assist-without-EAdi resembles auto-triggering with AI_Thille_. If not induced by backup modes during apnea, auto-triggering is another faulty condition where the ventilator triggers and cycles off uncontrollably, and hyperventilates the patient. Auto-triggering is a very difficult asynchrony to detect with AI_Thille_, because there is no true patient reference to validate the ventilator triggering [10].

To describe *dyssynchrony*, the method of Thille *et a*l. [1] involved detection of short and prolonged cycles (Table 1). Considering the natural variability in breathing, however, the significance of detecting these remains unclear [11,12]. The closest comparison to the NeuroSync index for short cycles would be late triggering and early cycling-off values, which would fall outside the box, that is, in the upper left quadrants in Figures 1 and 3. Long cycles (Figure 1 and Table 1) are likely to be associated with early trigger (lower quadrants) and/or delayed cycling-off (right-side quadrants) or repeated EAdi during assist (asynchrony).

The AI_Thille_ index [1] also includes double-triggering, an event corresponding to multiple-assist-during-EAdi with the NeuroSync index. Multiple-assist-during-EAdi reflects repeated trigger and cycling-off errors during the same neural effort, which are classified as dyssynchrony. It should be noted that in assist-volume control, double triggering is a severe asynchrony associated with excessive tidal volumes [13]. In non-flow and volume-regulated modes, double triggering would only cause a timing error with a short interruption of the inspiratory assist during an inspiratory effort.

The NeuroSync index also introduces another type of asynchrony labeled multiple-EAdi-during-assist, a severe type of asynchrony where the ventilator is delivering one breath for several neural inspiratory efforts. The AI_Thille_ has no counterpart for multiple-EAdi-during-assist.

Because EAdi-without-assist, assist-without-EAdi, and multiple-EAdi-during-assist all describe failures of the ventilator trigger and cycling-off functions, these events were labeled as 100% trigger error and 100% cycling-off error, and called asynchrony.

In the context of the above discussion it is important to note that AI_Colombo_[4] significantly increases the sensitivity to detect asynchrony compared to AI_Thille_.

Our results show that both the AI_Thille_ and the EAdi-verified AI_Colombo_[4] were not designed to detect timing errors between neural effort and assist-delivery (that is, they only detect asynchrony), whereas the NeuroSync index has added the ability to also quantify dyssynchrony and synchrony. This is evidenced in Figure 2A showing that the NeuroSync index reached 40% before AI_Colombo_ surpassed 10%. The close association between indices when AI_Colombo_ exceeds 10% (Figure 2B) shows that asynchronies are detected by both indices. As evidenced by a close relationship between the asynchronous events detected with the NeuroSync index and AI_Colombo_, most asynchronies defined by the NeuroSync index provide information similar to that of the pressure- and flow-based asynchrony index described by Thille *et al*. [1].

Another index of asynchrony based on EAdi was described by Beck *et al*. [14], where the sum of trigger delays and cycling-off delays (determined manually) were expressed as a percentage of the total neural respiratory cycle [8,14-16]. The NeuroSync index can be considered a development of the previously described EAdi-based index.

This new method has important clinical implications. The NeuroSync index provides real-time detection and quantification of: (i) patient ventilator asynchrony (of different types), (ii) dyssynchrony and (iii) synchrony. The method allows objective evaluation of patient neural breathing pattern and the ventilator performance, and could be used to adjust ventilator settings in order to optimize patient-ventilator interaction. Improved matching of patient and ventilator timings could enhance lung-distending pressure and ventilatory efficiency. Moreover, no study has yet shown any negative outcome of timing errors, related specifically to trigger and cycle-off delays (dyssynchrony). Asynchrony has been associated with prolonged time on mechanical ventilation [1,2]. The NeuroSync index, therefore, could provide a tool for future studies to determine acceptable limits of dyssynchrony. It should be pointed out that since the EAdi signal is a pneumatically independent signal, it is not affected by leaks, implying that the analysis presented could be applied reliably in intubated infants with uncuffed endotracheal tubes and during non-invasive ventilation.

## Conclusion

The present study introduces an automated method and the NeuroSync index to objectively determine patient-ventilator interaction with higher precision than previous methods. A dashboard style of graphical display allows a rapid overview of patient-ventilator interaction and breathing pattern.

## Key messages

• The diaphragm electrical activity is a useful signal for evaluating and monitoring patient-ventilator interaction.

• The NeuroSync index (an automated and standardized index to quantify patient-ventilator interaction) was found to be reproducible and correlated to manual analysis by experts.

• In mechanically ventilated adult patients, events can now be classified in an objective fashion as asynchronous, dyssynchronous or synchronous.

• The NeuroSync index determines patient-ventilator interaction with more sensitive analysis than previous methods.

• A dashboard style of graphical display allows a rapid overview and quantification of patient-ventilator interaction and breathing pattern at the bedside.

## Abbreviations

AI: _Colombo_ asynchrony index based on the definition of Colombo *et al*. [4]; AIThille: asynchrony index based on the definition of Thille *et a*l. [1]; EAdi: Electrical activity of the diaphragm; EAdiOFF: Manually or automatically determined 1/3 decrease in electrical activity of the diaphragm; EAdiON: Manually or automatically determined beginning of electrical activity of the diaphragm breath; ECG: Electrocardiograph; FN: Frequency of neural (patient) breaths; FV: Frequency of ventilator-delivered breaths; ICC: Intraclass correlation coefficient; NeuroSync: index index for patient-ventilator interaction based on automated algorithms described in the present paper; indexAUTO: Index for patient-ventilator interaction based on automated algorithms described in the present paper with automated selection of timings; NeuroSyncMANU index: Index for patient-ventilator interaction based on automated algorithms described in the present paper with manual selection of timings; PEEP: Positive end-expiratory pressure; PSOFF: End of pressure delivery; PSON: Start of pressure delivery; PV: Ventilator-delivered pressure.

## Competing interests

Drs. Beck and Sinderby have made inventions related to neural control of mechanical ventilation that are patented. The license for these patents belongs to Maquet Critical Care. Future commercial uses of this technology may provide financial benefit to Drs. Beck and Sinderby through royalties. Dr Beck and Dr Sinderby each own 50% of Neurovent Research Inc (NVR). NVR is a research and development company that builds the equipment and catheters for research studies. NVR has a consulting agreement with Maquet Critical Care. A Slutsky is a paid consultant to Maquet Critical Care.

## Authors’ contributions

CS conceived the study, described the algorithms for the analysis, and drafted the manuscript. SL performed data analysis and statistical analysis. DC collected the data, and revised the manuscript. GC collected the data and revised the manuscript. AS was involved with data interpretation and manuscript preparation. PN collected the data and revised the manuscript. JB participated in the design of the study and manuscript preparation. All authors read and approved the final manuscript.
